# State of the art review in gonadal dysgenesis: challenges in diagnosis and management

**DOI:** 10.1186/1687-9856-2014-4

**Published:** 2014-04-14

**Authors:** Bonnie McCann-Crosby, Roshanak Mansouri, Jennifer E Dietrich, Laurence B McCullough, V Reid Sutton, Elise G Austin, Bruce Schlomer, David R Roth, Lefkothea Karaviti, Sheila Gunn, M John Hicks, Charles G Macias

**Affiliations:** 1 Division of Pediatric Endocrinology, Baylor College of Medicine, Texas Children’s Hospital, Houston, TX 77030, USA; 2 Division of Pediatric and Adolescent Gynecology, Department of Obstetrics and Gynecology, Baylor College of Medicine, Texas Children’s Hospital, Houston, TX 77030, USA; 3 Center for Medical Ethics and Health Policy, Baylor College of Medicine, Texas Children’s Hospital, Houston, TX 77030, USA; 4 Department of Molecular and Human Genetics, Baylor College of Medicine, Texas Children’s Hospital, Houston, TX 77030, USA; 5 Division of Pediatric Urology, Department of Surgery, Baylor College of Medicine, Texas Children’s Hospital, Houston, TX 77030, USA; 6 Department of Pathology, Baylor College of Medicine, Texas Children’s Hospital, Houston, TX 77030, USA; 7 Evidence-Based Outcomes Center, Baylor College of Medicine, Texas Children’s Hospital, Houston, TX 77030, USA

**Keywords:** XY gonadal dysgenesis, Complete gonadal dysgenesis, Partial gonadal dysgenesis, Gonadectomy, Gonadal biopsy, Gonadoblastoma, Dysgerminoma, Carcinoma *in situ*, Malignancy risk, Ethics

## Abstract

Gonadal dysgenesis, a condition in which gonadal development is interrupted leading to gonadal dysfunction, is a unique subset of disorders of sexual development (DSD) that encompasses a wide spectrum of phenotypes ranging from normally virilized males to slightly undervirilized males, ambiguous phenotype, and normal phenotypic females. It presents specific challenges in diagnostic work-up and management. In XY gonadal dysgenesis, the presence of a Y chromosome or Y-chromosome material renders the patient at increased risk for developing gonadal malignancy. No universally accepted guidelines exist for identifying the risk of developing a malignancy or for determining either the timing or necessity of performing a gonadectomy in patients with XY gonadal dysgenesis. Our goal was to evaluate the literature and develop evidence-based medicine guidelines with respect to the diagnostic work-up and management of patients with XY gonadal dysgenesis. We reviewed the published literature and used the Grading of Recommendation, Assessment, Development, and Evaluation (GRADE) system when appropriate to grade the evidence and to provide recommendations for the diagnostic work-up, malignancy risk stratification, timing or necessity of gonadectomy, role of gonadal biopsy, and ethical considerations for performing a gonadectomy. Individualized health care is needed for patients with XY gonadal dysgenesis, and the decisions regarding gonadectomy should be tailored to each patient based on the underlying diagnosis and risk of malignancy. Our recommendations, based on the evidence available, add an important component to the diagnostic and management armament of physicians who treat patients with these conditions.

## Introduction

*Gonadal dysgenesis* is a term used for a unique subset of disorders of sexual development (DSD) [1] characterized by incomplete or defective formation of the gonads (ovary or testis) due to either structural or numerical anomalies of the sex chromosomes or mutations in the genes involved in the development of the gonad [2]. Dysgenetic gonads are characterized by variable degrees of immaturity or dysfunction, which can manifest in a wide range of genital ambiguity. Gonadal dysgenesis can be classified as either complete (CGD) or partial (PGD) depending on the gonadal morphology [3,4]. In CGD (i.e., 46,XY Swyer syndrome), no gonadal development occurs, and, as a consequence, patients have a completely female phenotype due to the lack of any gonadal steroid production. In PGD in which a Y chromosome is present, there is incomplete testis determination and the external phenotype depends on the degree of testicular function. The most common karyotype seen in PGD is 45,X/46,XY, but 46,XY and other forms of mosaicism involving the Y chromosome also can be seen.

Patients with gonadal dysgenesis who have a Y chromosome or Y-chromosome material are at increased risk for developing germ cell tumors such as gonadoblastoma or carcinoma *in situ* (CIS), with the potential for malignant transformation to dysgerminoma or seminoma, respectively [5-7]. The term *gonadoblastoma* was first introduced by Scully in 1953 and is the most common germ cell tumor seen in patients with XY gonadal dysgenesis [8]. A benign germ cell ovarian neoplasm composed of germ cells and sex cord stromal cells, a gonadoblastoma almost always arises from a dysgenetic gonad with a Y chromosome [9]. Gonadoblastoma usually presents in the second decade, but cases occurring in early infancy have been reported [10]. In 50-60% of cases, gonadoblastomas are associated with malignant germ cell tumors, most commonly dysgerminomas. The prognosis is favorable when the gonadoblastoma is associated with dysgerminoma, but unfavorable when associated with other germ cell tumors including yolk sac tumors, seminomas, immature teratomas, embryonal carcinomas, or choriocarcinomas [11]. CIS, otherwise known as *intratubular germ cell neoplasia unclassified*, is the common precursor for testicular germ cell tumors including seminomas, embyronal carcinomas, teratomas, and yolk cell tumors [6]. Gonadal dysgenesis is a known risk factor for CIS [12]. The natural history of untreated CIS is a 40% estimated risk of progression to invasive cancer within three years and a 50% estimated risk of progression within five years [13].

To prevent the development of malignancy in patients with XY gonadal dysgenesis, gonadectomy typically is recommended, but debate ensues concerning which patients require surgery and the appropriate timing [14]. Further, no standard approach or guidelines have been established for the diagnostic workup and management of these patients. The objective of this paper was to review the existing evidence and to provide recommendations for the appropriate diagnostic work-up and timing of performing a gonadectomy in the patient with XY CGD or XY PGD. We reviewed which patients require gonadectomy, factors involved in the risk of developing a malignancy, and ethical considerations with respect to gonadectomy. We used the Grading of Recommendation, Assessment, Development, and Evaluation (GRADE) system when appropriate to grade the evidence and provide recommendations. The GRADE system is an evidence-based medicine tool used to evaluate the quality of the evidence and the strength of recommendations [15]. We provide herein both a review of the literature and guidelines for endocrinologists, gynecologists, ethicists, psychologists, urologists, and geneticists who care for patients with XY gonadal dysgenesis, with the intent that this approach will be relevant for the standardization of the field in the upcoming years.

## Methods

We identified two clinically relevant questions to be answered from the evidence for diagnosis and management of patients with XY CGD or XY PGD:

1. In patients with suspected XY gonadal dysgenesis, *what diagnostic testing* should be considered to establish the diagnosis?

2. Which patients with XY gonadal dysgenesis require gonadectomy, and what is the appropriate timing?

Sub-questions identified were:

a. What are differences in risks of malignancy based on diagnoses?

b. Is there a role for gonadal biopsy?

c. What ethical considerations must be taken into account before undertaking a gonadectomy?

To answer these questions, databases were searched for research-based articles on infants, children, and adults with XY CGD or XY PGD. The databases included Pub Med, Cochrane Collaboration, and Google Scholar. We included only articles published in English and no earlier than 1970, as prior to this date the literature in this area consists of mainly case reports and no large case series. Specific keywords and terms used included: complete or pure gonadal dysgenesis, mixed or partial gonadal dysgenesis, XY gonadal dysgenesis, diagnosis, gonadectomy, gonadoblastoma, dysgerminoma, malignancy risk, gonadal biopsy, and ethics.

We searched the literature specifically for articles that addressed each question. The GRADE system was used when applicable. The quality of the evidence was evaluated as “very-low quality”, “low quality”, “moderate quality”, or “high quality”. The recommendations provided were either “strong” or “weak”. For questions for which the GRADE system did not apply, a consensus statement was formulated.

## Evidence and recommendations

Our search of the major databases yielded articles addressing each question. Overall, the search yielded consensus statements, observational studies, case reports, personal experience, and review articles. No randomized controlled trials were identified. The evidence and recommendations for each question are described below:

### Question 1: in patients with suspected XY gonadal dysgenesis, *what diagnostic testing* should be considered to establish the diagnosis?

#### *Evidence*

**Figure 1:**
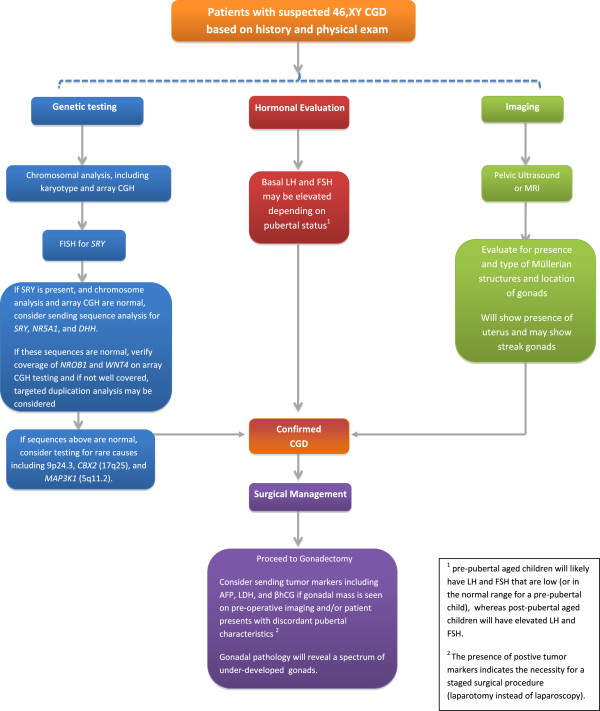
Diagnostic work-up for patients with 46,XY CGD.

**Figure 2:**
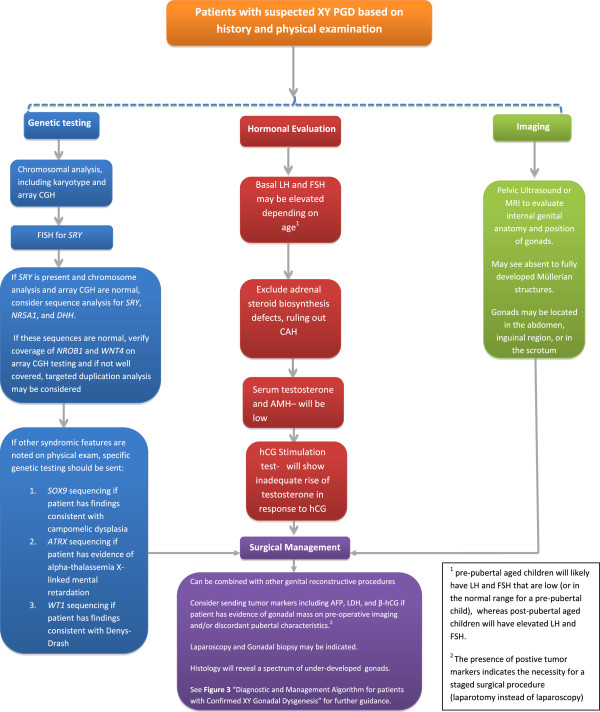
Diagnostic work-up for patients with XY PGD.

The search yielded three review articles and several case reports that provided recommendations for the diagnostic work-up of gonadal dysgenesis. The review articles were by Ostrer [3], Fleming and Vilain [16], and Michala and Creighton [17]. Because they are review articles, the GRADE tool could not be applied. According to these reviews, the diagnosis of XY gonadal dysgenesis is established based on physical examination, hormonal evaluation, imaging studies, genetic studies including karyotype, and gonadal histology (see discussion below and Figures 1 and 2). As there are differences between the clinical findings in XY CGD and XY PGD, each will be discussed separately.

#### *Complete (pure) XY gonadal dysgenesis (XY CGD)*

Patients with 46,XY CGD, or Swyer syndrome, are phenotypically female with normal Müllerian structures and bilateral streak gonads [18]. They most commonly present in adolescence with delayed puberty or primary amenorrhea due to their non-functional gonads. Physical exam reveals normal female external genitalia. Endocrine evaluation usually shows hypergonadotropic hypogonadism with elevated basal LH and FSH, as the gonads are not functional. Imaging studies, including pelvic ultrasound or MRI, demonstrate the presence of a uterus and may show bilateral streak gonads. If gonadectomy or gonadal biopsy is performed, gonadal histology reveals the presence of bilateral dysgenetic streak gonads. Tumor markers including AFP, β-hCG, and LDH are known to be associated with germ cell malignancy. Although the evidence for routinely sending serum tumor markers for screening purposes in patients with XY CGD is lacking, positive tumor markers in the setting of a gonadal mass on pre-operative imaging and/or discordant pubertal characteristics (i.e., precocious puberty or virilization) suggests that a staged surgical procedure is necessary [19]. Finally, chromosomal analysis reveals a 46,XY karyotype. Mutations and deletions in the *SRY* (sex-determining gene on the Y chromosome) have been reported in the literature to account for 10-20% of the cases of 46,XY CGD [17,20]. Other mutations identified have included *NR5A1* (9q33) [16,20,21], *DHH* (12q13.1) [3,20], *NROB1* (DAX 1) [3,16], *WNT4 *[3,16], *DMRT1* (9p24.3) deletion [3,16,22], *CBX2* (17q25) deletion [23], and a heterozygous mutation in *MAP3K1* (5q11.2) [24]. In many cases, the cause of XY CGD remains unknown.

#### *Recommendations*

For patients with suspected XY CGD, we recommend the following testing to establish the diagnosis (see Figure 1):

a. Physical examination: including genitourinary exam to assess for clitoromegaly, phallic size, hypospadias, presence of palpable gonads either in the labioscrotal fold or inguinal region and to evaluate the introitus and patency of the vagina and for other dysmorphic features/malformations that may indicate an underlying syndrome.

b. Genetic testing (adapted from Ostrer [3]):

i. Chromosomal analysis, including karyotype and array CGH

ii. FISH for *SRY*. Patients with Turner’s syndrome and a 45,X karyotype require FISH for *SRY* to evaluate for cryptic Y mosaicism [32].

iii. If *SRY* is present and chromosome analysis and array CGH are normal, consider sequence analysis for *SRY*, *NR5A1*, and *DHH*. If these sequences are normal, verify coverage of *NROB1* and *WNT4* on array CGH testing and if not well covered, consider targeted duplication analysis.

iv. If other syndrome features are noted on examination, specific genetic testing should be sent

1. *SOX9* sequencing if patient has findings consistent with campomelic dysplasia

2. *ATRX* sequencing if patient has evidence of alpha-thalassemia X-linked mental retardation

3. *WT1* sequencing if patient has findings consistent with Denys-Drash syndrome

c. Hormonal evaluation

i. Basal LH and FSH

ii. Serum testosterone levels

iii. hCG stimulation test

iv. Anti-Müllerian Hormone

v. Exclude adrenal steroid biosynthesis defects

d. Imaging: pelvic ultrasound or MRI to look for internal genital anatomy and gonad position; may not be able to visualize undescended gonads on imaging.

e. Surgical management: laparoscopy and gonadal biopsy may be indicated. If the patient has a gonadal mass on pre-operative imaging and/or discordant pubertal characteristics, consider serum tumor markers, including AFP, LDH, and beta-hCG, for preoperative planning. If tumor markers are positive, a staged surgical procedure (laparotomy instead of laparoscopy) is indicated.

#### *Evidence quality: low*

*Strength of Recommendation*: *Strong* for all categories except sections iii and iv of the genetic testing section and routinely sending serum tumor markers, which are weak recommendations.

#### *Partial XY gonadal dysgenesis (PGD)*

XY PGD includes a heterogeneous group of individuals with varying degrees of clinical phenotypes and various karyotypes. Included in this group are patients with Turner syndrome who have a mosaic karyotype, usually 45,X/46,XY. The most common karyotype of patients with XY PGD is 45,X/46,XY, but others may have 46,XY or 45,X/47,XYY. Patients can have a spectrum of presentations, including females with a Turner syndrome phenotype, ambiguous genitalia, under-virilized males, or normal phenotypic males [25]. Phenotypically normal males with 45,X/46,XY may not be diagnosed unless they are evaluated in adulthood for infertility secondary to reduced sperm production from dysgenetic testes [26]. Imaging shows absent to fully developed Müllerian structures, depending on the degree of testicular dysgenesis. Gonadal histology may reveal either bilateral dysgenetic testes or one streak gonad and a contralateral dysgenetic or normal-appearing testis. As seen in patients with XY CGD, patients with XY PGD often show evidence of hypergonadotropic hypogonadism with elevated basal LH and FSH levels at the age when puberty normally occurs. Patients with PGD have been shown to have a diphasic pattern of LH and FSH secretion whereby gonadotropin concentrations are significantly elevated during infancy, fall to nearly normal values during childhood, and return to significantly elevated levels after the normal age of puberty [27,28]. Measurements of serum testosterone and anti-Müllerian hormone (AMH) usually are decreased, and human chorionic gonadotropin (hCG) stimulation testing usually shows minimal to no elevation in testosterone levels in response to hCG. The evidence for routinely sending serum tumor markers such as AFP, LDH, and beta-hCG for screening purposes in patients with XY PGD is lacking. As discussed for XY CGD, positive tumor markers in the setting of a gonadal mass on pre-operative imaging and/or discordant pubertal characteristics would suggest that a staged surgical procedure is necessary [19]. Mutations have been described in *SRY *[3,16], *NR5A1*(9q33) [29,30], *DHH* (12q13.1) [3], *NROB1* (DAX 1) [3,16], and *WNT4 *[3,16].

Several disorders, in addition to Turner syndrome, are associated with XY PGD. Campomelic dysplasia is a skeletal malformation syndrome caused by mutations in *SOX9 *[3,16]. Denys-Drash syndrome includes mesangial sclerosis of the kidney and Wilms tumor caused by *WT1* germline mutations [3,16]. Frasier syndrome, also caused by *WT1* germline mutations, is associated with 46,XY CGD and involves focal and segmental glomerulosclerosis of the kidney [3,16]. Alpha-thalassemia/X-linked mental retardation syndrome (ATRX) is characterized by mental retardation, often associated with α-thalassemia and gonadal abnormalities such as undescended testicles, testicular dysgenesis, and ambiguous external genitalia [3,16,31].

#### *Recommendations*

For patients with suspected XY PGD, we recommend the following for establishing the diagnosis (see Figure 2):

a. Physical examination: including genitourinary exam to assess for clitoromegaly, phallic size, hypospadias, presence of palpable gonads either in the labioscrotal fold or inguinal region and to evaluate the introitus and patency of the vagina and for other dysmorphic features/malformations that may indicate an underlying syndrome.

b. Genetic testing (adapted from Ostrer [3]):

i. Chromosomal analysis, including karyotype and array CGH

ii. FISH for *SRY*. Patients with Turner’s syndrome and a 45,X karyotype require FISH for *SRY* to evaluate for cryptic Y mosaicism [32]

iii. If *SRY* is present and chromosome analysis and array CGH are normal, consider sequence analysis for *SRY*, *NR5A1*, and *DHH*. If these sequences are normal, verify coverage of *NROB1* and *WNT4* on array CGH testing and if not well covered, consider targeted duplication analysis.

iv. If other syndrome features are noted on examination, specific genetic testing should be sent

1. *SOX9* sequencing if patient has findings consistent with campomelic dysplasia

2. *ATRX* sequencing if patient has evidence of alpha-thalassemia X-linked mental retardation

3. *WT1* sequencing if patient has findings consistent with Denys-Drash syndrome

c. Hormonal evaluation

i. Basal LH and FSH

ii. Serum testosterone levels

iii. hCG stimulation test

iv. Anti-Müllerian Hormone

v. Exclude adrenal steroid biosynthesis defects

d. Imaging: pelvic ultrasound or MRI to look for internal genital anatomy and gonad position; may not be able to visualize undescended gonads on imaging.

e. Surgical management: laparoscopy and gonadal biopsy may be indicated. If the patient has a gonadal mass on pre-operative imaging and/or discordant pubertal characteristics, consider serum tumor markers, including AFP, LDH, and beta-hCG, for preoperative planning. If tumor markers are positive, a staged surgical procedure (laparotomy instead of laparoscopy) is indicated.

#### *Evidence quality: low*

*Strength of Recommendation: Strong* for all categories except section iii of the genetic testing section, and routinely sending serum tumor markers, which are weak recommendations.

### Question 2: which patients with XY gonadal dysgenesis require gonadectomy, and what is the appropriate timing?

#### *Evidence*

Thirteen observational studies from 1970–2013 were identified that provided information about indications for performing gonadectomy and/or recommendations regarding timing for performing gonadectomy in patients with XY gonadal dysgenesis [9,20,33-43]. Only studies with more than 10 patients were included for review. We used the GRADE tool to evaluate the quality of the evidence and provide recommendations. The studies are summarized in Table 1.

**Table 1:** GRADE evaluation of literature for timing of gonadectomy

**Study**	**Type of study**	**Diagnoses**	**Location of gonads that show malignancy: if specified**	**Conclusions from each study regarding timing of gonadectomy**	**Design limitations**
**Wunsch, et al. 2012 **[33]	Observational Cohort study	8 patients with CGD underwent gonadectomy:	All patients with CGD had intra-abdominal gonads	Early gonadectomy for patients with CGD	Small sample size, lack of blinding, lack of allocation concealment
		-Ages ranged from 1–25 years	All patients with PGD had intra-abdominal streak gonads.	For patients with PGD and non-scrotal gonads, early gonadectomy may be warranted	
		-3 patients (37.5%) had evidence of *in situ neoplasia* (ages 3, 12, 18);			
		-2 of these patients also had dysgerminoma. 12 patients with PGD had gonadal tissue evaluation:			
		1 patient (8.3%) had gonadoblastoma at age 6			
**Johansen, et al. 2012 **[34]	Observational Retrospective study	15 patients with PGD (45X/46,XY and variants) had gonadal samples for review:	14 year old male had left inguinal dysgenetic testis with CIS	No specific recommendations for timing, does indicate that CIS originates before puberty	Small sample size, lack of blinding, lack of allocation concealment, ascertainment bias
		-3 patients (20%) had evidence of *In situ Neoplasia:*	2 year old male with right inguinal dysgenetic testis		
			4 year old female with left inguinal dysgenetic testis		
		-2 males (ages 2 and 14) and 1 female (age 4) had CIS			
**Martinerie, et al. 2012 **[35]	Observational Retrospective Study	20 boys with PGD (45,X/46,XY) were studied	13 year old with intra-abdominal streak gonad	No specific recommendations for timing of gonadectomy.	Small sample size, lack of blinding, lack of allocation concealment
		-2 patients (10%) had evidence of malignancy:	23 year old with intrascrotal dysgenetic testis (inguinal at birth with orchidopexy at 9 years of age).	Recommend strict surveillance of gonads and testicular function in patients with PGD raised as males	
		Dysgerminoma found in a 13 year old male.			
		Seminoma found in a 23 year old male.			
**Rocha, et al. 2011**[20]	Observational Retrospective study	9 patients with XY CGD who had histology available.	Abdominal	Recommend gonadectomy at diagnosis	Limited sample size, lack of blinding, lack of allocation concealment
		-Gonadoblastoma in 4 patients (44%) ages 14–17, Two of which also had dysgerminoma (22%)			
**Cools, et al. 2011**[36]	Observational study	Obtained 84 gonadal samples from 39 patients with PGD who were 45,X/46, XY:	1 patient with mild undervirilization had right abdominal gonad with gonadoblastoma (age not specified)	In females with PGD, tumor risk is limited but gonads are not functional, making gonadectomy the most reasonable option.	Small sample size, lack of blinding, lack of allocation concealment, selection bias (no gonadal tissue from undiagnosed 45,X/46,XY males).
		-*In Situ* Neoplasia found in 4 different patients (10.2%).	1 patient with ambiguous phenotype had left abdominal gonad with gonadoblastoma (age 1)	Malignancy risk in males appears inversely related to degree of virilization (more virilized, less risk).	
		-3 patients had gonadoblastoma, 1 had CIS.	1 patient with ambiguous phenotype had dysgenetic inguinal testis with gonadoblastoma (age 1)	Low threshold for gonadectomy in males with ambiguous genitalia.	
			1 patient with female phenotype had right abdominal gonad with CIS (age 16)	For mildly undervirilized males: 1 prepubertal biopsy and 1 post-pubertal biopsy	
**Michala, et al. 2008 **[37]	Observational Retrospective study	Gonadal histology reviewed in 22 patients with Swyer syndrome:	Abdominal	Recommend bilateral gonadectomy as soon as diagnosis is made	Limited sample size, retrospective study
		-45% with germ cell tumors;			
		-32% with dysgerminoma (ages 10–31 years)			
		-14% with gonadoblastoma (ages 17, 19, and 27 yrs)			
**Cools, et al. 2006 **[14]	Observational Retrospective Study	60 gonadectomy samples from 43 patients with gonadal dysgenesis (included CGD and PGD):	Did not specify gonadal location	Gonadal histology revealing undifferentiated gonadal tissue or testicular tissue staining positive for OCT3/4 on the basal lamina contains high risk for gonadal tumors and should lead to immediate gonadectomy.	Small sample size, lack of blinding, lack of allocation concealment
		-35% incidence of germ cell tumors in patients with GD (n = 16), ages ranging from 4 months-25 yrs).		Testicular tissue displaying maturation delay of germ cells can be left in situ, given that its localization allows for adequate follow-up.	
		-All but 1 patient with malignancy had Y chromosome material.		Ovarian tissue can be safely left in place	
		-Invasive germ cell tumors found in 13% (n = 6)		Streak is not functional, making its preservation controversial	
**Mazzanti, et al. 2005 **[38]	Observational Study	Identified 14 Turner patients with Y-chromosome material:	Abdominal	Recommend bilateral gonadectomy for all Turner patients with Y chromosome material	Limited sample size, lack of blinding, lack of allocation concealment.
		-12 out of 14 patients consented to gonadectomy:			

		33% of gonadectomized patients had gonadoblastoma (ages 2,7,11, 15 yrs)			
		The 15 year-old patient also had a immature teratoma, and a endodermal sinus tumor			
**Slowikowska-Hilczer, et al. 2003 **[39]	Observational Study	Gonadal histology reviewed in 40 cases of gonadal dysgenesis:	All gonads were located in the abdomen or upper segment of the inguinal canal	No specific recommendations for timing of gonadectomy	Limited sample size, lack of blinding, lack of allocation concealment
		-67.5% had 46,XY Karyotype and the remainder had numerical and structural abberations of sex chromosomes.			
		One patient with 46,XY karyotype had seminoma from abdominal gonad (age 17)			
		CIS present in 14 patients (35%) with GD			
		Sex cord-derived tumors including gonadoblastoma nests and unclassified mixed germ cell-sex cord-stromal tumors were present in 11 patients (27.5%) with GD			
		Ages of malignancy ranged from 7 months to 19 years			
**Mendes, et al. 1999 **[40]	Observational study	36 patients with Turner syndrome were studied:	Abdominal	Recommend gonadectomy in Turner Syndrome patients who are Y positive	Limited sample size, lack of blinding, lack of allocation concealment
		Two patients were found to be Y positive by PCR			
		Of the two Y-positive patients, one had gonadoblastoma (50%)			
**Gourlay, et al. 1994 **[42]	Observational Retrospective Study	11 patients with PGD had gonadal tissue for review:	All but 1 patient with PGD and malignancy had abdominal gonads	Recommend early gonadectomy in all patients with XY gonadal dysgenesis as tumors can develop at an early age	Limited sample size, lack of blinding, lack of allocation concealment
		Six patients (54%) had germ cell tumors; ages ranging from 1 month to 19 years	1 PGD patient (age 19) with a seminoma had scrotal gonads		
		One patient with 46,XY CGD had a gonadoblastoma (age 17)	The patient with CGD had abdominal gonads		
**Robboy, et al. 1982 **[43]	Observational Retrospective Study	Obtained gonadal tissue from 21 patients with PGD:	53 year old with gonadoblastoma had abdominal gonads	Recommend early gonadectomy	Limited sample size, lack of blinding, lack of allocation concealment
		-Three patients (14.2%) with XY PGD had malignancy:	One patient had a gonadoblastoma and seminoma in a scrotal-inguinal gonad 15 years after the contralateral testis was removed (age not specified)		
		Two patients with XY PGD had gonadoblastomas and one of these was overgrown by a seminoma.	2 week old with seminoma had an abdominal gonad		
		One patient with XY PGD had seminoma (age 2 weeks)			
**Scully, et al. 1970 **[9]	Observational Retrospective Study	Reviewed clinical characteristics of 74 cases of gonadoblastoma:	Majority were abdominal gonads	Recommend early gonadectomy	Lack of blinding, lack of allocation concealment
		25 phenotypic females, 35 virilized females, 13 phenotypic males.	Inguinal gonadoblastomas were seen in several of the phenotypic males (exact number not specified)		
		43 patients had invasive germinoma			
		Ages ranged from 1 to 38 years			
		Karyotypes were available in 30/74 patients:			
		57% had 46,XY karyotypes			
		30% with 45,X/46,XY karyotype			
		3% (1 patient) with 45,X karyotype			
		10% with other forms of mosaicism			

#### *Complete XY gonadal dysgenesis (XY CGD)*

Several studies specifically address timing of gonadectomy in patients with XY CGD (Swyer syndrome) [20,33,37]. In these studies, the incidence of gonadal malignancy in patients with XY CGD ranged from 37.5%-45%. Of those patients with XY CGD who had gonadal malignancy, dysgerminoma was present in 22-66%. The majority of cases of gonadoblastoma or dysgerminoma are discovered at the time the diagnosis of XY CGD is established, which typically occurs in adolescence although cases of malignancy identified in young children have been reported. In the studies reviewed in Table 1, the youngest patient with dysgerminoma was 10 years old and the youngest patient with gonadoblastoma was 3 years old [33,37]. The consistent recommendation in the literature is for bilateral gonadectomy to be performed as soon as possible once the diagnosis of XY CGD (Swyer syndrome) is established, given the high risk of gonadoblastoma with progression to dysgerminoma.

#### *Partial XY gonadal dysgenesis (XY PGD)*

Inconsistency occurs in the literature with respect to timing of gonadectomy in patients with XY PGD. As discussed earlier, XY PGD includes a heterogeneous group of individuals with various degrees of clinical phenotypes and karyotypes, with the most common karyotype being 45,X/46,XY. In earlier literature, early gonadectomy typically was recommended in patients with XY PGD to prevent development of malignancy, although some authors recommended waiting until the age of puberty as the risk of malignancy prior to that time was acceptably low [44]. More recent studies suggest a more individualized and conservative approach in the decision-making process for gonadectomy by taking into account certain factors including location of the gonads (abdominal, inguinal, or scrotal), internal and external phenotype, and sex of rearing. In the studies reviewed in Table 1, most of the cases of malignancy in XY PGD occurred in gonads that were located intra-abdominally, followed by inguinal gonads. Very few cases of intra-scrotal malignancy in patients with XY PGD were reported. As seen in Table 1, all three of the XY PGD patients with malignancy originating from scrotal gonads had a seminoma and were discovered in the second and third decades of life. Given this observation, in patients with XY PGD who are reared as males, many studies recommend surveillance of the gonads with a low-threshold for gonadectomy in those who have non-scrotal gonads, an ambiguous phenotype, or insufficient testicular function. Several authors have recommended regular testicular self-examination and yearly testicular ultrasound in those patients with XY PGD who are reared as males. Testicular biopsy as a means for surveillance has been discussed in the literature and will be reviewed in a following section.

The literature yields no unified approach with respect to timing of gonadectomy in XY PGD patients who are reared as females. Most of the studies addressing XY PGD patients have looked specifically at 45,X/46,XY mosaic Turner patients. In the studies that are reviewed in Table 1, the risk of malignancy in female patients with 45,X/46,XY karyotype ranged from 2.2-50%, with gonadoblastoma presenting as early as 2 years of age. Although most of the studies recommend early gonadectomy in patients with 45,X/46,XY Turner syndrome, a recent study by Cools, et al., [36] suggested that girls without signs of virilization have a low risk of developing a tumor (2.2% with malignancy in their series), so gonadectomy could be postponed in patients who are reluctant to have surgery. This recommendation should be taken with caution, as other studies have shown higher rates of malignancy in this patient population, and there are no established guidelines for monitoring these patients for development of a malignancy if they choose to forego gonadectomy.

#### *Recommendations (See Figure* 3*)*

**Figure 3:**
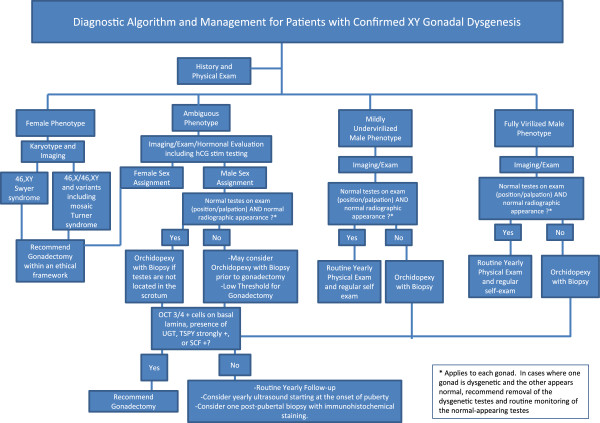
Diagnostic algorithm and management for patients with confirmed XY gonadal dysgenesis.

a. We recommend that patients with XY CGD (i.e., Swyer syndrome) have bilateral gonadectomy at the time of diagnosis to prevent development of gonadal malignancy.

#### *Evidence quality: low*

Strength of recommendation: strong

b. We recommend that patients with XY PGD with nonscrotal gonads that cannot be repositioned surgically into a scrotal position have bilateral gonadectomy.

#### *Evidence quality: low*

Strength of Recommendation: Strong

c. We suggest that patients with XY PGD with scrotal gonads being reared as males undergo routine monitoring with self-examination for development of malignancy.

#### *Evidence quality: low*

Strength of recommendation: weak

### 2a: what are differences in risks of malignancy based on diagnoses?

#### *Evidence*

The articles in the medical literature that addressed this question are primarily review articles, rendering the GRADE tool not applicable. A recent review by Cools, et al. [14] reported the overall prevalence of germ cell tumors in patients with gonadal dysgenesis as 12%. The prevalence may be underestimated because untreated patients were not included and the presence of a Y chromosome was not an inclusion criterion for many studies. Risks for developing malignancies have been noted based on gonadal dysgenesis etiology (PGD vs. CGD), gonad location, degree of virilization, and certain tumor marker expression in gonadal tissue.

For patients with 46,XY CGD (Swyer syndrome), the risk of developing gonadal malignancy has been reported to be 15-35% [14,20,45]. A recent study by Michala, et al., reported the prevalence of germ cell tumors to be as high as 45% [37]. Given this high risk of malignancy in patients with XY CGD, the recommendation to remove gonads at the time of diagnosis is definitely justified.

The risk of developing gonadal malignancy in patients with PGD who have 45,X/46,XY and variants is reported by Cools, et al., as 15-40% [14]. In patients with mixed gonadal dysgenesis or asymmetrical gonadal differentiation, the estimated tumor prevalence is reported to be approximately 15%, although this figure may be an underestimation [14]. Overall, it is in concordance with the reported malignancy prevalence in the studies summarized in Table 1, with malignancy risk ranging from 8.3-54% in patients with XY PGD. A series by Gravholt, et al., [46] in 2000 examined the prevalence of gonadoblastoma in Y-positive Turner patients and reported it to be 7-10%, which is lower than reported in other published studies. Although this risk is significant, the authors argued that in situations in which patients or parents do not wish to proceed with gonadectomy, routine monitoring with ultrasound may be used to evaluate for development of malignancy. No evidence supports appropriate frequency of monitoring or suggests that other methods of monitoring, such as laboratory screening, may be more useful in detecting onset of malignancy.

Recent studies have suggested that a correlation exists between the degree of virilization of the external genitalia and gonadal function with subsequent risk for developing a malignancy [34,36]. In a study by Cools, et al., [36], the risk of developing a tumor was associated significantly with the clinical phenotype and was found to be greatest (52%) in those with ambiguous genitalia. The location of the gonads also plays a role in the development of malignancy. In the studies summarized in Table 1, most malignant tumors occurred in gonads in the abdomen; however, several cases of inguinal or scrotal testis showed evidence of either pre-malignant precursor lesions or *in situ* neoplasia.

Certain immunohistochemical markers (OCT 3/4, c-KIT, TSPY, VASA) have been identified that can be useful in establishing the diagnosis of malignant germ cell tumors [14,41,45]. Of these, the combination of OCT 3/4 and TSPY appears to be the most robust in identifying germ cell tumors [41]. OCT 3/4 is a transcription factor that is present during fetal gonadal development but is not normally present postnatally. The location of OCT 3/4-positive cells plays an important role in the risk of developing a malignancy. Cools, et al., showed that OCT 3/4-positive cells positioned along the basal lamina of the seminiferous tubule have an increased risk for malignant transformation, whereas OCT 3/4-positive cells located more centrally in the seminiferous tubules reflected a delay in maturation and were not associated with an increased risk for malignancy [47]. The *TSPY* gene (testis-specific protein-Y) is thought to be a main candidate gene involved in development of a gonadoblastoma, and its expression confers an increased risk of malignancy [14].

Many factors must be considered for each individual patient when assessing the risk for developing a malignancy. Table 2, adapted from Plescakova, et al., [45] displays malignancy risk stratification based upon virilization, location of gonads, pathologic features, and immunohistochemical marker findings.

**Table 2:** Malignancy risk based upon type of gonadal dysgenesis, location of gonad, gross pathology, and immunohistochemistry

	**Low risk**	**Intermediate risk**	**High risk**
**Degree of virilization**	Normally virilized males	Mild undervirilization	Ambiguous genitalia
**Location of gonad**	Scrotal gonad with normal appearance	Inguinal gonad	Abdominal gonad
**Gross pathology**	-Streak gonad without germ cells	Dysgenetic testicle that is OCT 3/4 positive with intermediate risk criteria	-Undifferentiated gonadal tissue
	-Ovary		
	- Testicle (OCT 3/4 negative)		
	- Dysgenetic testicle that is OCT 3/4 negative		-Dysgenetic testicle that is OCT 3/4 positive with high risk criteria
**Immunohistochemistry**	OCT 3/4 negative	OCT 3/4 positive cells located luminally, scattered within the whole gonad, TSPY negative or weakly positive, SCF negative, age < 1 year.	OCT 3/4 positive cells located in the basal lamina, focal location, TSPY strongly positive, SCF positive, age > 1 year.

Adapted from Pleskacova, et al. [45].

### 2b: is there a role for gonadal biopsy?

#### *Evidence*

Five observational studies were published from 1985 to 2013 that assisted in answering the question concerning gonadal biopsy [33,36,42,48,49]. The GRADE tool was used to evaluate the quality of the evidence and to provide recommendations. These studies are summarized in Table 3.

**Table 3:** GRADE evaluation of literature for use of gonadal biopsy

**Study**	**Type of study**	**Summary of findings**	**Conclusions from each study regarding use of gonadal biopsy**	**Design limitations**
Farrugia, et al. 2013 [48]	Observational Retrospective study	Histology of 46 gonads from patients with 45,X/46,XY or 45,X/47,XYY PGD was reviewed.	In patients raised as males, where dysgenetic testes are retained, biopsy at orchidopexy and also post-pubertal with immunohistochemical staining (OCT 3/4 and TSPY) is recommended	Limited sample size, lack of blinding, lack of allocation concealment
		Does not specify who had biopsy vs. gonadectomy.		
		No evidence of malignancy in any patient		
Wunsch, et al. 2012 [33]	Observational Cohort study	6 out of 12 patients with mixed or partial GD had biopsy to evaluate for malignancy (no gonadectomy).	Biopsy can be used for early diagnosis of germ cell tumors and follow-up.	Limited sample size, lack of blinding, lack of allocation concealment
		1 patient found to have tubular in situ neoplasia.		
Cools, et al. 2011 [36]	Observational Study	Histology of 87 gonads from patients with 45,X/46,XY was reviewed.	For mildly undervirilized males, recommend 1 prepubertal biopsy and 1 post-pubertal biopsy.	Limited sample size, lack of blinding, lack of allocation concealment
		Biopsy was done in 15 patients.		
		All of the tumors in this series were in situ germ cell neoplastic lesions, discovered after prophylactic gonadectomy.	In patients with ambiguous genitalia, biopsy can be used to asses tumor risk, but low threshold for gonadectomy	
		No tumors identifed in the patients who had gonadal biopsy alone.		
Gourlay, et al. 1994 [42]	Observational Retrospective Study	Reviewed pathology from 21 patients with DSD who underwent bilateral gonadectomy at time of diagnosis.	Gonadal biopsy is unreliable in excluding the presence of small tumors	Limited sample size, lack of blinding, lack of allocation concealment
		Pathology revealed many different combinations of testis, ovary, streak, and tumor within the same individual gonad.		
Müller, et al. 1985 [49]	Observational Study	Gonadal tissue from multiple scrotal or labial gonadal biopsies was studied in 4 patients with 45,X/46,XY GD (ages 1 month to 18 years)	Biopsy of scrotal gonads should be done at time of diagnosis of GD to exclude presence of tumor.	Limited sample size, lack of blinding, lack of allocation concealment
		All 4 patients had evidence of CIS:		
		-2 patients had CIS on initial biopsy	In boys without signs of CIS on initial biopsy, repeat biopsy should be performed after puberty because prepubertal CIS lesions may be missed.	
		-2 patients had CIS only on repeat biopsy (8 months and 16 years)		

The few studies that address this question in the literature have differing viewpoints on the usefulness of gonadal biopsies in patients with XY gonadal dysgenesis. In several studies, gonadal biopsy is suggested as a useful technique for early diagnosis of germ cell tumors and for follow-up. Additionally, some studies have suggested using laparoscopic gonadal biopsy in cases with unclear diagnoses, allowing for histological examination of gonadal tissue prior to proceeding with gonadectomy [33]. Gonadal biopsy appears to be most useful in monitoring for tumor development in mildly undervirilized males with testes that are either located in the scrotum, or can be brought down surgically into the scrotum. Several recent papers recommend that in patients with XY PGD and a male phenotype, one pre-pubertal biopsy, typically in combination with orchidopexy, and one post-pubertal biopsy with appropriate immunohistochemical staining, including OCT3/4 and TSPY, are warranted to identify patients at risk for malignancy [36,48]. The evidence is limited for the usefulness of gonadal biopsy to assess tumor risk in patients with female or ambiguous phenotypes, as their risk for developing gonadal malignancy is high, and the threshold to perform gonadectomy in these patients is low.

Several limitations of gonadal biopsy must be taken into consideration. The retrospective study by Gourlay, et al., [42] noted that gonadal tumors can easily be missed on biopsy because of the many different combinations of cells (testicular, ovarian, fibrous, and tumor) that may be found within the same individual gonad, as well as the limited sampling and sampling errors. Therefore, they reported that gonadal biopsy may be unreliable in excluding the presence of small tumors. Müller, et al., [49] also demonstrated that premalignant lesions may be seen on repeat biopsies from patients with XY PGD who initially had normal gonadal biopsies. It is important to note that there are no prospective studies that show the usefulness of gonadal biopsy in early detection of malignancy or improving outcomes. Given this, patients with XY PGD who undergo gonadal biopsy should be followed and outcomes should be reported.

#### *Recommendations (see Figure* 3*)*

a. *In patients with XY CGD*, gonadal biopsy has no role, as these patients ultimately require gonadectomy to prevent development of a malignancy.

#### *Evidence quality: low*

Strength of recommendation: strong

b. *In patients with XY PGD* who are reared as males with mild undervirilization and gonads that can be repositioned into the scrotum via orchidopexy, we recommend one prepubertal gonadal biopsy at the time orchidopexy is performed and a post-pubertal gonadal biopsy to monitor for malignancy. If both testes are located inguinally, both should be biopsied. If one testis is located inguinally and one is located in the scrotum and appears normal, consider biopsy of both testes. Given this recommendation, clinicians must be aware that, due to limited sampling and sampling error, a normal gonadal biopsy does not completely rule out the presence of a small tumor.

#### *Evidence quality: low*

Strength of recommendation: weak

c. *In patients with XY PGD* and are phenotypically normal males with normal appearing testicles that are located in the scrotum, we do not recommend gonadal biopsy but do recommend routine testicular self-examinations.

#### *Evidence quality: low*

Strength of recommendation: weak

### 2c: what ethical considerations must be taken into account before undertaking gonadectomy?

#### *Evidence*

Three articles from 2005 to 2010 provided ethical recommendations for surgical interventions in patients with DSD [50,51]. A summary of these articles is presented in Table 4. Because these are review articles, the GRADE tool was not used. There are no outcome studies that address this question. These articles emphasized that interventions with irreversible consequences such as gonadectomy must be performed based upon a compelling medical indication following thorough diagnostic evaluation. A multidisciplinary team, including specialists in endocrinology, urology, gynecology, psychology, and ethics, should be involved in the decision-making process. The authors noted that the decision for surgical intervention must take into account the best interest of the patient and should also include the family in the decision-making process. If interventions are not urgent, they should be delayed until the child is old enough to make an informed decision. On the other hand, if the decision is made to refrain from an irreversible intervention, this decision should also be justified with appropriate evidence.

**Table 4:** Summary of literature addressing ethical recommendations for surgical intervention in DSD patients

**Study**	**Type of study**	**Considerations specific to gonadectomy**	**Relevant ethical dilemmas/principles identified**	**Recommendations**
**Gillam, et al. 2010 **[51]	Review	Early Gonadectomy:	1. Psychological issues poorly understood	1. Improve understanding physical and psychological dilemmas facing each patient
		1. Medical indication (i.e. hernia)	2. No guarantee of adult gender identity	2. Thorough informed consent process
		2. Parental concerns about malignancy	3. Surgical decision making places pressure on parents, who may be incompletely informed	3. Referral to multi-disciplinary team – and if not available refrain from any potentially harmful practice or surgery
		3. Difficulty accepting phenotype without surgery/improved psychological outcome		
		Late Gonadectomy – after puberty completed		
		No Gonadectomy:		
		1. Long term follow up required		
**Wiesemann, et al. 2010 **[50]	Review	“Unless well-being would otherwise be severely impaired, decisions about removal of organs or structures important to… physical integrity or sexual identity (such as gonads) should be left up to the affected persons themselves”	1. Secrecy within families, lack of informed consent and adolescent assent	1. Acknowledge that even a participant child cannot act in their future self’s best interest, only in the current best interest
			2. conflict between the interests of a child and the interests of the future adult	2. In the absence of an objective best interest for the child in managing DSD, parents should play a major role in decision making, on a case-by-case basis
			3. conflict between right to familial privacy and state’s interest in protecting the child	3. We should not make a sweeping recommendation on the timing of a surgical intervention in the absence of medical necessity
			4. immediacy of health threat	4. Involvement of the child at a developmentally appropriate level
			5. child’s right to dignity and bodily integrity	5. Allowing the adult patient to access all past medical records
				6. Careful documentation of outcomes for future information
**Maharaj, et al. 2005 **[52]	Review	1.Paper addresses only infants and young children	1. Minimizing physical risk to child	1. Act in the best interests of the child, taking account wishes of the parents
		2. Unclear how to decide whether it is worse to be at future risk of malignancy or risk of distress in the future from gonadectomy	2. Minimizing psychosocial risk to child	2. In situations that are complex with no clear best answer, where future outcomes are difficult to predict, parents’ wishes should be respected
		3. Fertility potential may be a factor, including presence and functionality of gonads, or presence/functionality of other reproductive organs. May conflict with another risk, such as future malignancy.	3. Preserving potential for fertility	3. None of the principles should be considered to outweigh the others and must be appropriately balanced
		4. Acknowledging that there may be medical advances in the future which could allow fertility even in apparent non-functional gonadal tissue	4. Preserving or promoting capacity to have satisfying sexual relation	4. Ethical decision-making in this field should be approached systematically and in a multi-disciplinary fashion
			5. Leaving options open for the future	
			6. Respecting parents’ wishes and beliefs	

Given the irreversible nature of gonadectomy, certain ethical considerations must be taken into account in addition to determining each individual patient’s risk for developing a malignancy. Both risks and benefits are involved in either retaining or removing gonads, and a general, beneficence-based principle of intervening only when the benefits are reliably judged to outweigh the risks should be maintained [50-52]. Benefits of undergoing a gonadectomy would include decreasing the risk of developing a gonadal malignancy. In the case of a patient with XY PGD who is assigned a female sex, the function of gonads at puberty may cause unwanted virilization, rendering a gonadectomy psychosocially beneficial. In contradistinction, certain benefits may be associated with retaining the gonad. Surgical procedures can lead to associated morbidity, and for situations with lower risks of development of a malignancy, it may be reasonable to wait until the patient has reached the capacity for developmentally appropriate assent or can legally consent before being subjected to such risk [51,52]. In addition, for patients with XY PGD and a male sex assignment, the gonads may have partially functioning testicular tissue that could be a source of hormone production through puberty and potential fertility. Overall, the decision for performing a gonadectomy must be made on a case-by-case basis based on the best interest of the patient.

#### *Recommendations*

a. The decision for gonadectomy should be made on a case-by-case basis, in conjunction with a multidisciplinary team and the family.

#### *Evidence quality: low*

Strength of recommendation: strong

b. Gonadectomy may be considered **only** if the benefits clearly outweigh the risks.

#### *Evidence quality: low*

Strength of recommendation: strong

## Conclusions

Using a systematic approach in evaluating the literature allowed us to develop recommendations for the diagnostic work-up, assessment of gonadal malignancy risk, timing of gonadectomy, and ethical considerations that must be incorporated when providing care for patients with XY gonadal dysgenesis. We chose to focus on these particular aspects of gonadal dysgenesis as they can be used to assess the risk of developing a gonadal malignancy and are areas in which no standardized approach has been established. We have provided an algorithm for diagnostic work-up and gonadal malignancy risk stratification based on the existing published evidence in this field. Our review is limited due to the paucity of long-term outcome studies and no randomized controlled studies in the area of XY gonadal dysgenesis. Although this paucity of information does contribute to a low quality of evidence available for review, we have found that strong recommendations exist for many of the questions addressed in this paper. Further studies are necessary to assess the risk for developing malignancy and to evaluate various interventions in affected patients. In conclusion, this paper illustrates that patients with XY gonadal dysgenesis require personalized health care and that the decision for performing a gonadectomy should be tailored to each individual patient based on the underlying specific clinical and histopathologic diagnosis and risk for malignancy. Our recommendations contribute important components that augment the diagnostic and management armament employed by physicians who treat patients with these conditions.

## Abbreviations

CGD: Complete gonadal dysgenesis; PGD: Partial gonadal dysgenesis; MGD: Mixed gonadal dysgenesis; CIS: Carcinoma *in situ*; SCF: Stem cell factor; TSPY: Testis-specific protein-Y; DSD: Disorders of sex development; UGD: Undifferentiated gonadal tissue.

## Competing interests

The authors involved in this paper declare that they have no competing interests.

## Author’s contributions

BMC performed the literature review, used the GRADE tool to evaluate the literature, and drafted the manuscript. RM assisted with literature review, helped draft the manuscript, and made key changes to the intellectual content. JD, LM, RS, EA, BS, DR, SG, and MJH critically reviewed the manuscript and made key changes with respect to the design and intellectual content. LK was involved in the initial conception and design of the manuscript as well as critical review and key changes to the intellectual content. CM provided the training necessary for composing an evidence-based medicine article, critically appraised the paper and provided key changes to the intellectual content. All authors read and approved the final manuscript.

## Author’s information

BMC is a second year pediatric endocrinology fellow at Baylor College of Medicine, Texas Children’s Hospital.

RM is a second year pediatric & adolescent gynecology fellow at Baylor College of Medicine, Texas Children’s Hospital.

JD is an associate professor, chief, and fellowship director of pediatric & adolescent gynecology at Baylor College of Medicine, Texas Children’s Hospital.

LM is a professor of medicine and medical ethics, and is the chair of the center for medical ethics and health policy at Baylor College of Medicine.

RS is an associate professor of the department of molecular and human genetics at Baylor College of Medicine, genetics residency program director, and medical director of the biochemical genetics laboratory at Baylor College of Medicine.

EA is a genetic counselor in the department of molecular and human genetics at Baylor College of Medicine.

BS is an assistant professor of urology at Baylor College of Medicine, Texas Children’s Hospital.

DR is a professor and chief of pediatric urology at Baylor College of Medicine, Texas Children’s Hospital.

LK is a professor of pediatric endocrinology & metabolism at Baylor College of Medicine, Texas Children’s Hospital.

SG is an associate professor of pediatric endocrinology & metabolism at Baylor College of Medicine, Texas Children’s Hospital.

MJH is a professor of pathology at Baylor College of Medicine, Texas Children’s Hospital.

CM is an associate professor in the department of pediatric emergency medicine at Baylor College of Medicine, Texas Children’s Hospital. CM is also the director for the Evidence based Outcomes Center and Center for Clinical Effectiveness at Texas Children’s Hospital.
